# Supplementary material for: Toxicity in Goats Exposed to Arsenic in the Region Lagunera, Northern Mexico
Source: Vet Sci. 2020 May 4;7(2):59. doi: 10.3390/vetsci7020059 (PMC7357139; doi:10.3390/vetsci7020059)

## Slide 1
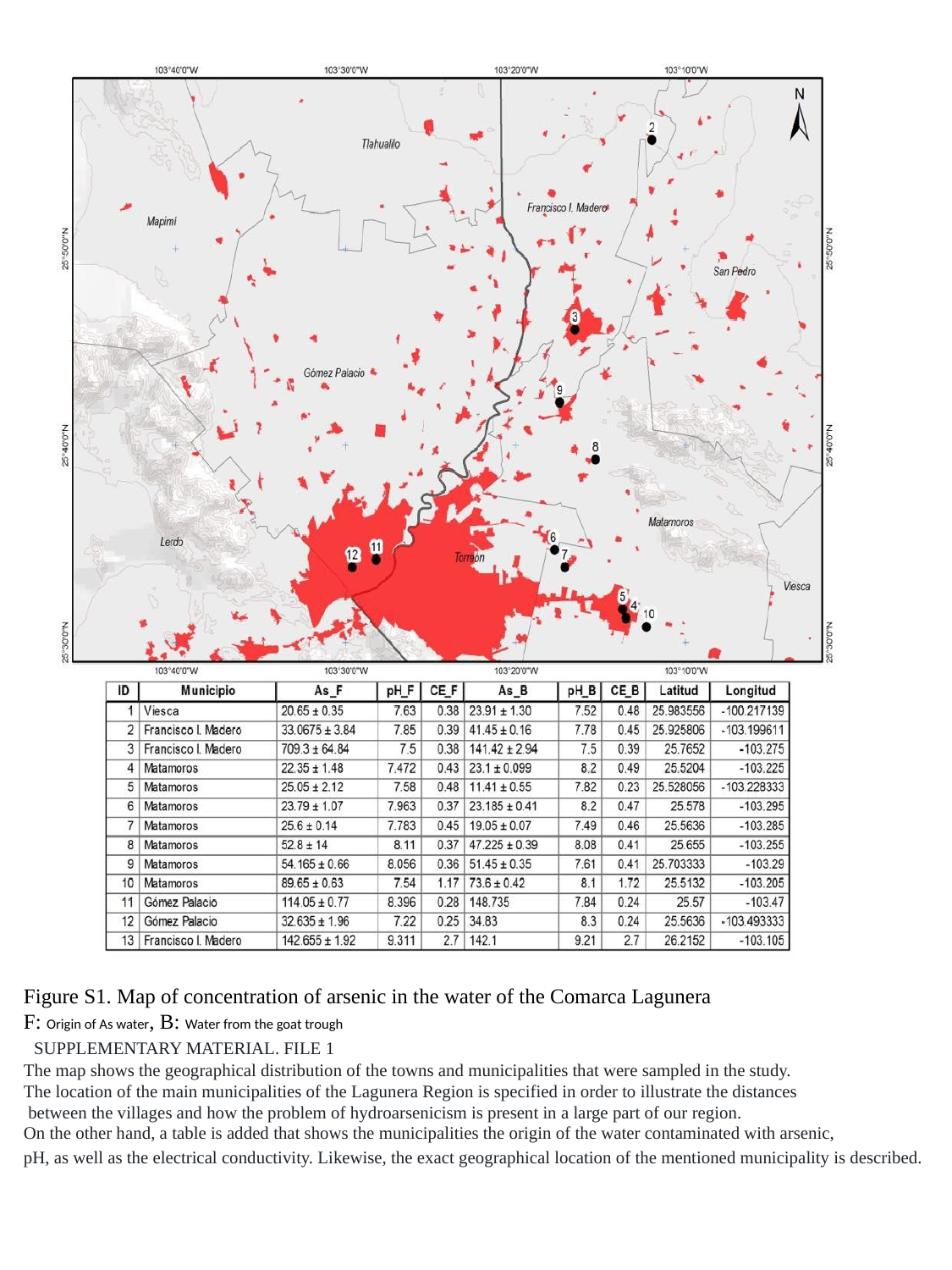

Figure S1. Map of concentration of arsenic in the water of the Comarca Lagunera
F: Origin of As water, B: Water from the goat trough
  SUPPLEMENTARY MATERIAL. FILE 1
The map shows the geographical distribution of the towns and municipalities that were sampled in the study.
The location of the main municipalities of the Lagunera Region is specified in order to illustrate the distances
 between the villages and how the problem of hydroarsenicism is present in a large part of our region.
On the other hand, a table is added that shows the municipalities the origin of the water contaminated with arsenic,
pH, as well as the electrical conductivity. Likewise, the exact geographical location of the mentioned municipality is described.

## Slide 2
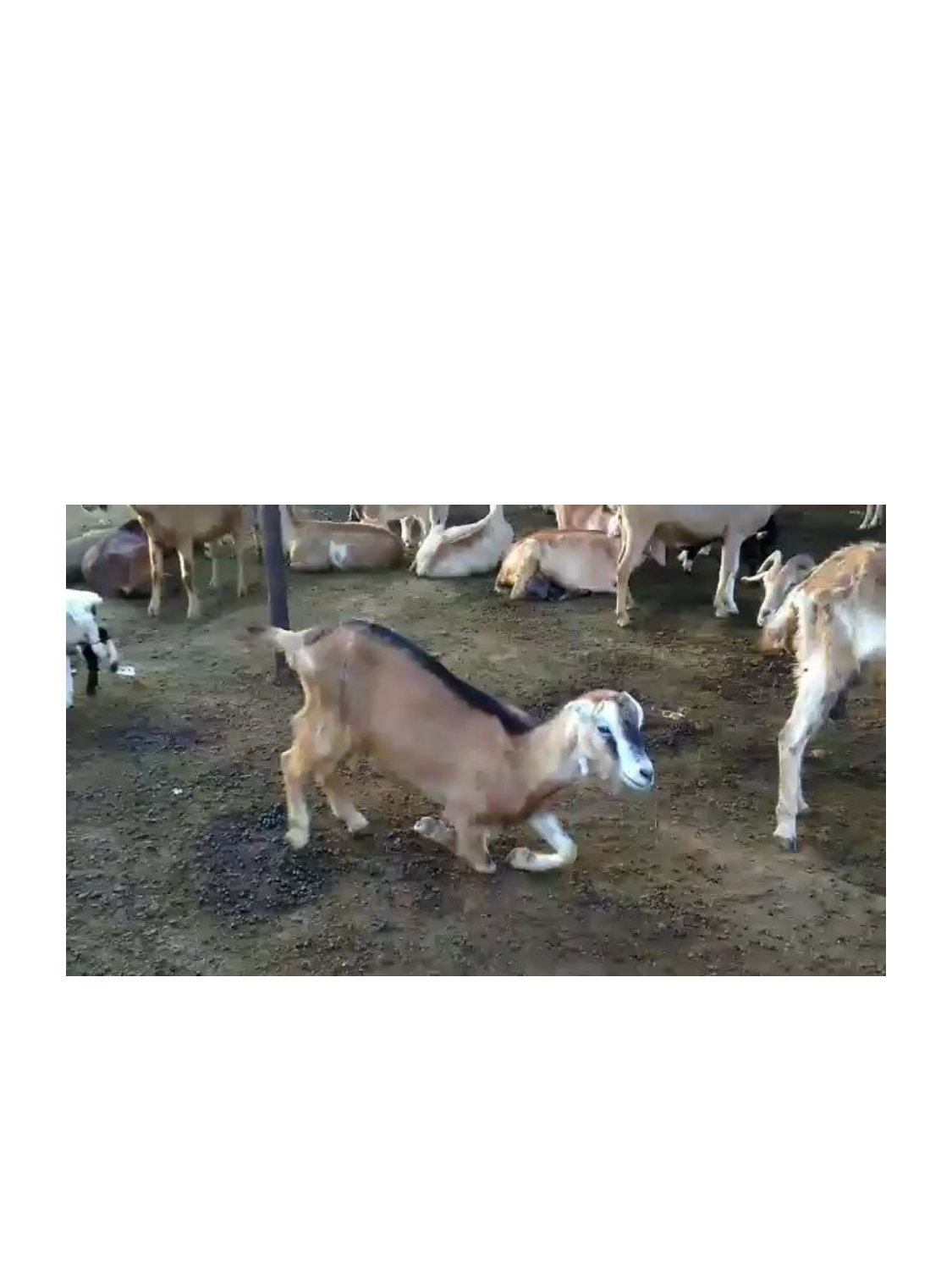

## Slide 3
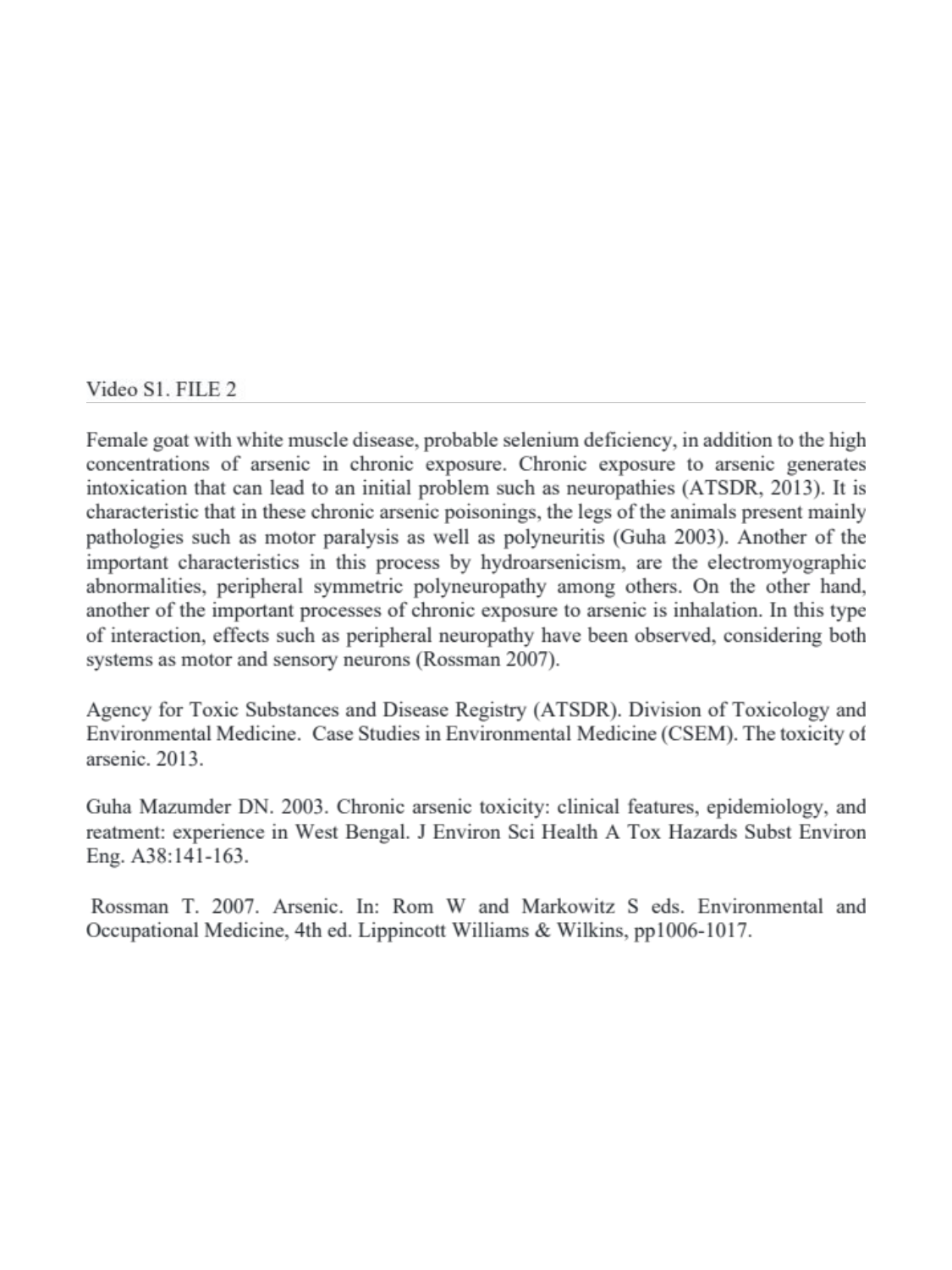

Supplement: Supplementary file 1 [file vetsci-07-00059-s001.zip › vetsci-770457-supplementary.pptx]
